# Pulmonary Vein Thrombosis and Pulmonary Embolus in a Pregnant Patient: A Case Report

**DOI:** 10.7759/cureus.40538

**Published:** 2023-06-16

**Authors:** Daniel J Berman, Melissa N Jordan

**Affiliations:** 1 Emergency Medicine, Naval Medical Readiness Training Center Okinawa, Fleet Post Office, USA; 2 Emergency Medicine, Naval Medical Readiness Training Center San Diego, San Diego, USA

**Keywords:** pregnancy complication, pregnancy, idiopathic pulmonary vein thrombus, submassive pulmonary embolism, case report, idiopathic pulmonary vein thrombosis, saddle pulmonary embolus, pulmonary vein thrombosis

## Abstract

We report a case of a pregnant woman who presented to the emergency department complaining of dyspnea and syncope and was ultimately diagnosed with pulmonary vein thrombosis and a saddle pulmonary embolus on computed tomography pulmonary angiography. Proper identification is critical for prompt management to avoid significant life-threatening sequela.

## Introduction

Pulmonary vein thrombosis (PVT) is a rare but serious disease process most often seen postoperatively in cases of lung transplantation, lobectomy, and radiofrequency catheter ablation for atrial fibrillation [1,2]. It is also seen in patients with hematologic disorders such as sickle cell disease or primary lung malignancies [1]. Idiopathic cases of PVT are extremely rare and limited to case reports [3]. The presentation of PVT is often nonspecific and may include symptoms such as a cough, dyspnea, and pleuritic chest pain [1,2]. To our knowledge, this report is the first case of PVT in a pregnant patient, as well as the first case to describe both PVT and pulmonary embolism (PE) in the same patient.

## Case presentation

A 38-year-old woman, 20 weeks pregnant, was brought in by emergency medical services to the emergency department for a sudden onset of dyspnea and a brief episode of syncope. On scene, she was hypoxic to 88% on the pulse oximeter and in mild respiratory distress.

The patient reported a previous episode approximately one week prior, which took 20-30 minutes to resolve. However, she did not seek care at that time. She reported a distant history of anemia and was taking 81 mg of aspirin for her moderate-risk pregnancy given her age of over 35 and first pregnancy. She otherwise reported taking prenatal vitamins, iron supplementation, and ascorbic acid. She denied a history of prolonged immobilization, travel, or a previous history of venous thromboembolism. She denied hitting her head or abdomen or having any chest pain, abdominal pain, or vaginal bleeding at the time. She did report a maternal history of lower extremity deep vein thrombosis.

The patient’s initial vitals were a pulse of 138 beats per minute, a blood pressure of 92/48 mm Hg, a respiratory rate of 28 breaths per minute, an oxygen saturation of 94% on six liters of nasal cannula, and an oral temperature of 37.3 degrees Celsius. On physical examination, she was diaphoretic and anxious. There were no signs of external trauma on the initial assessment. Cardiac auscultation revealed tachycardia without murmurs, rubs, or gallops. Lung sounds were clear throughout; however, she was in mild respiratory distress, evident by her tachypnea. An abdominal exam revealed a soft, non-tender, gravid uterus.

**Figure 1:**
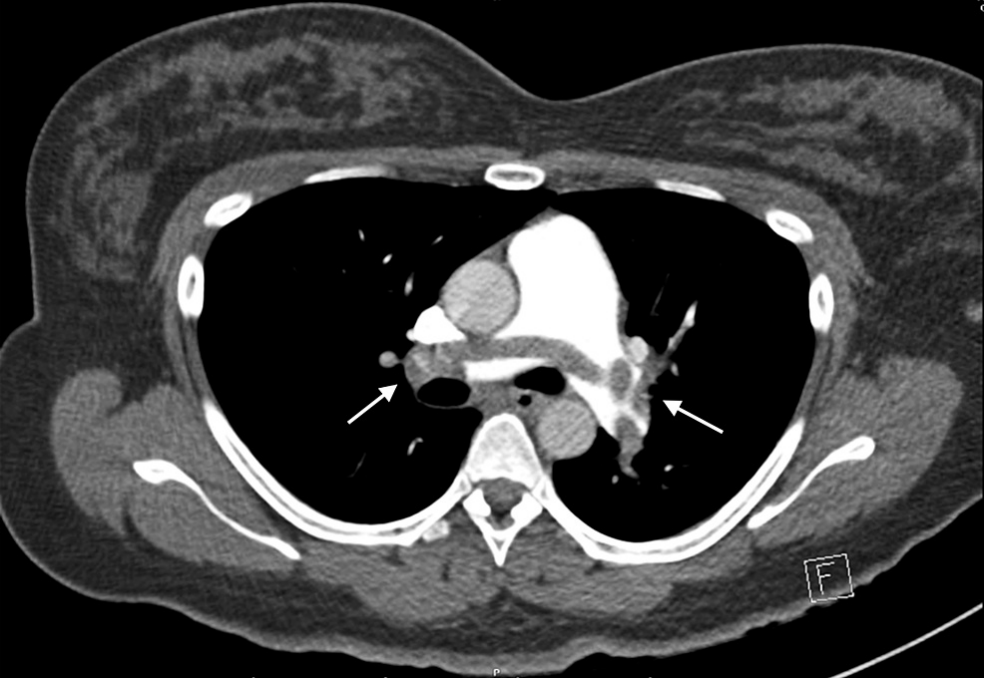
Axial images of the CTA of the chest with saddle pulmonary embolus (arrows)

Bedside ultrasonography revealed normal lung sliding bilaterally without any lung abnormalities over the anterior lung fields and a single intrauterine pregnancy consistent with the stated gestational age. A limited bedside echocardiogram revealed septal bowing and enlargement of the right ventricle. A computed tomography angiogram (CTA) of the chest revealed extensive pulmonary embolism burden with evidence of right heart strain, most notably a saddle pulmonary embolus (Figure 1).

**Figure 2:**
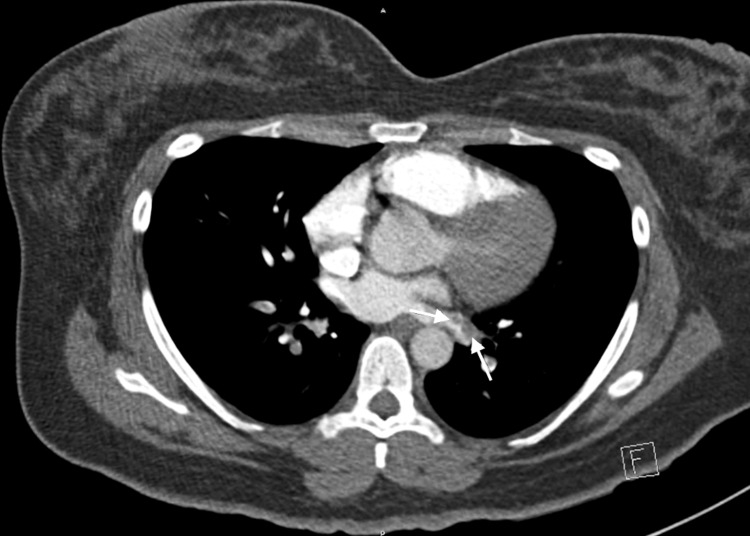
Axial image of the CTA of the chest with pulmonary vein thrombosis (arrows)

In addition to the PE, the CTA revealed a left inferior pulmonary vein thrombus without extent to the left atrium (Figure 2). The COVID-19 polymerase chain reaction was negative.

Relevant laboratory values showed a mild elevation in troponin T and pro-brain natriuretic peptide (Table 1).

**Table 1:** Blood serum test results ng/mL: nanograms per milliliter; pg/mL: picograms per milliliter

Blood serum test	Results	Normal values
Troponin T	0.029 ng/mL	<0.02 ng/mL
Pro-brain natriuretic peptide	254 pg/mL	<100 pg/mL

## Discussion

PVT can be difficult to identify both clinically and radiographically. Some etiologies that can predispose patients to develop PVT are post-operative: following lung transplants or lobectomies, status post-radiofrequency ablation for atrial fibrillation, or primary lung malignancies [1,2]. It can also be seen in hypercoagulable states, such as those seen in malignancies, COVID-19 patients, or those with hematologic pathologies such as sickle cell trait [1,3,4].

Clinical presentations can be very non-specific and include dyspnea, hemoptysis, fevers, chest pain, and hypoxemia [1-5]. Patients with severe disease can develop sequelae of right heart failure and pulmonary hypertension, even predisposing them to systemic thrombotic emboli, with the possibility of large thrombi extending into the left heart or systemic embolization causing a cerebrovascular accident [1,2,4]. Chronic or more insidious patient presentations have been associated with pulmonary edema and fibrosis [2]. Acute cases of PVT have been seen in patients who presented with massive hemoptysis and decompensated heart failure [6,7].

Diagnostic modalities for the identification of pulmonary vein thrombi include magnetic resonance with IV contrast or computed tomography arthrography. An echocardiogram, specifically a transesophageal echocardiogram, is also a useful adjunct to evaluate the pulmonary veins [1,2,4,5]. Severe disease may have ultrasound evidence of right heart failure and pulmonary edema [1,2]. Our patient had a mixed picture, as she had both PVT and a saddle PE with evidence of right heart strain.

The goals of treatment are to reverse the underlying etiology of the PVT and use systemic anticoagulation to decrease the risk of systemic embolization. Individual cases may require thrombectomy or pneumonectomy. Furthermore, antibiotics should be considered for infarcted tissue at risk for infection [1]. Once the PVT is identified and addressed, there can be rapid resolution with an improved clinical picture [2].

## Conclusions

The patient was administered an 80-unit/kg IV bolus of heparin, followed by a continuous heparin infusion at 18 units/kg/hr, and admitted to the intensive care unit for further management by maternal-fetal medicine and pulmonary/critical care.

The patient remained without neurologic sequela from her PVT and was discharged home after five days of inpatient admission on subcutaneous enoxaparin. She was asymptomatic and did not require supplemental oxygen. Her pregnancy status remained unchanged, and she had a normal anatomy scan while inpatient.
